# Structure Collisions between Interacting Proteins

**DOI:** 10.1371/journal.pone.0019581

**Published:** 2011-06-02

**Authors:** Dorothea Emig, Oliver Sander, Gabriele Mayr, Mario Albrecht

**Affiliations:** Department of Computational Biology and Applied Algorithmics, Max Planck Institute for Informatics, Saarbrücken, Germany; Koç University, Turkey

## Abstract

Protein-protein interactions take place at defined binding interfaces. One protein may bind two or more proteins at different interfaces at the same time. So far it has been commonly accepted that non-overlapping interfaces allow a given protein to bind other proteins simultaneously while no collisions occur between the binding protein structures. To test this assumption, we performed a comprehensive analysis of structural protein interactions to detect potential collisions. Our results did not indicate cases of biologically relevant collisions in the Protein Data Bank of protein structures. However, we discovered a number of collisions that originate from alternative protein conformations or quaternary structures due to different experimental conditions.

## Introduction

Most molecular processes involve interactions between proteins. The physical contact between protein interaction partners is formed at defined binding interfaces, and one protein may bind various interaction partners at the same interface or at different interfaces. Due to the increasing number of protein structures available in the Protein Data Bank (PDB) [1], systematic protein interaction studies integrating structural information have become more and more attractive [2], [3], [4], [5].

It has been a commonly accepted assumption that a protein containing multiple, non-overlapping interfaces can always interact simultaneously with other proteins. As part of a large-scale structural analysis of a protein interaction network in yeast, Kim and colleagues presumed that the number of simultaneous interactions a protein can participate in is determined by the number of its non-overlapping binding interfaces [6]. To this end, the authors gave a structure-based definition of single- and multi-interface proteins and found differences in expression profiles and evolutionary rates. Subsequently, Kim *et al.* investigated the role of disorder in structural networks and discovered that disordered interface regions are more common in single-interface proteins [7]. Other studies also included structural information into their systematic analyses to increase the informative value of a given network or the reliability of protein interaction predictions [8], [9].

Further protein network analyses concentrated on various aspects of single- and multi-interface proteins, ranging from protein interaction partners to interface specificity and interaction motifs. For instance, Keskin and Nussinov studied multi-specific interfaces known to bind proteins with different structures [10]. They primarily focused on the ability of one binding interface to form interactions with different proteins and identified key residues potentially responsible for binding. In a related study, Humphris and Kortemme analyzed restrictions imposed on the protein sequences for permitting multiple binding partners and predicted residues essential for the respective interactions [11]. Aragues and colleagues analyzed hub proteins, i.e., highly connected proteins, in the context of interaction motifs (iMotifs) [12] and compared their results to those previously found by Kim *et al.* [6]. The iMotif approach is based on the idea that proteins sharing interaction partners most likely interact with them via the same binding sites. Clustering proteins according to their interaction partners showed that the number of iMotifs correlated with the number of protein interfaces in the work by Kim *et al*. [6]. Aragues and coworkers also found that cellular essentiality and gene conservation correlate better with the number of interacting motifs than with the absolute number of interactions. Furthermore, Tuncbag *et al.* presented a concept integrating the time dimension into protein interaction networks using protein structures and interface information, which was utilized for the characterization of interactions in the p53 pathway [13]. This work highlights the fact that the formation of simultaneous protein interactions depends on various factors including temporal aspects, which should be considered in the analysis of protein interaction networks.

**Figure 1:**
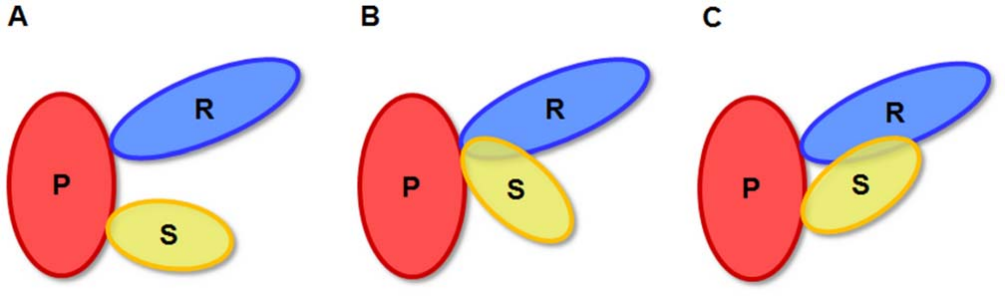
Schematic overview of structurally possible interactions between three proteins P, R and S. (A) The three proteins interact simultaneously via two distinct binding interfaces at P. (B) R and S cannot interact with P at the same time due to the overlapping binding interface at P. (C) Although R and S interact with P via separate binding interfaces, their simultaneous interaction with P is prevented by a collision of R and S.

To our knowledge, however, the above-described basic assumption has never been investigated that simultaneous interactions at different interfaces are always spatially possible. In detail, two or more binding partners *R* and *S* of a protein *P* might collide in three-dimensional (3D) space, which would prevent the simultaneous interaction of *R* and *S* with *P* even though the binding sites are non-overlapping (Figure 1). Therefore, we developed a structure collision approach for interactions between protein structure chains in the Protein Data Bank (PDB) to examine spatial conflicts between interaction partners.

## Materials and Methods

In this study, we investigated whether a protein *P* can simultaneously bind two different proteins *R* and *S* at distinct binding interfaces. We refer to protein *P* as the *primary protein*, while its interaction partners *R* and *S* are the *secondary proteins*. In principle, we regarded all known protein structures that contain an interaction between proteins *P* and *R* in one structure and between proteins *P* and *S* in another structure, requiring that *R* and *S* were bound to *P* at different interfaces. After the two primary proteins *P* of the pairwise protein interactions *P-R* and *P-S* were superimposed, a collision detection method was applied to identify structure collisions between simultaneously possible interactions of the three proteins (Figure 1).

In detail, we first retrieved all protein structure files from the PDB [1]. In case of NMR entries, we used the first model since it is regarded as the representative protein structure according to the PDB instructions. We identified the binding interface residues between all pairs of interacting protein structure chains by means of the SPPIDER web service (http://sppider.cchmc.org/) [14]. Then we annotated all PDB chains with UniProtKB accession numbers using the mapping provided by PDBSWS [15]. We used the resulting annotations to identify pairs of protein interactions *P-R* and *P-S*, where the UniProtKB accession numbers of the primary protein *P* were identical for both interactions while the UniProtKB accession numbers of the secondary proteins *R* and *S* were different.

We compared the binding interface residues of each protein interaction pair to find pairs with overlapping or distinct interfaces. The binding interfaces of *P* in the interaction pair *P-R* and *P-S* were defined to be distinct if all interface residues in *P-R* were different from those in *P-S* (analogous to the study by Kim *et al.* [6]). If at least one interface residue was involved in both interactions, we regarded the interface as overlapping and the simultaneous interaction of the three proteins as impossible. This definition is intentionally strict to exclude any potential overlap of the binding interfaces since we want to detect solely collisions of proteins *R* and *S* that have clearly disjunct binding interfaces. To further ensure that the proteins can really establish a functional interaction, we considered only those interaction pairs *P-R* and *P-S* whose number of interface residues for each interaction was at least five residues.

After all pairs of interactions *P-R* and *P-S* that met the described criteria were identified, the primary proteins were superimposed and tested for collisions between the secondary proteins. Even if the UniProtKB accession numbers of two PDB chains are identical, the actual structure may not contain the complete protein because certain protein regions might not have been structurally determined. Therefore, the primary proteins *P* had to be aligned with each other to identify their corresponding PDB residues for computing the transformation matrix of the superposition. The alignments were performed using ClustalW [16], and the resultant files were parsed to extract the matching PDB residues.

**Figure 2:**
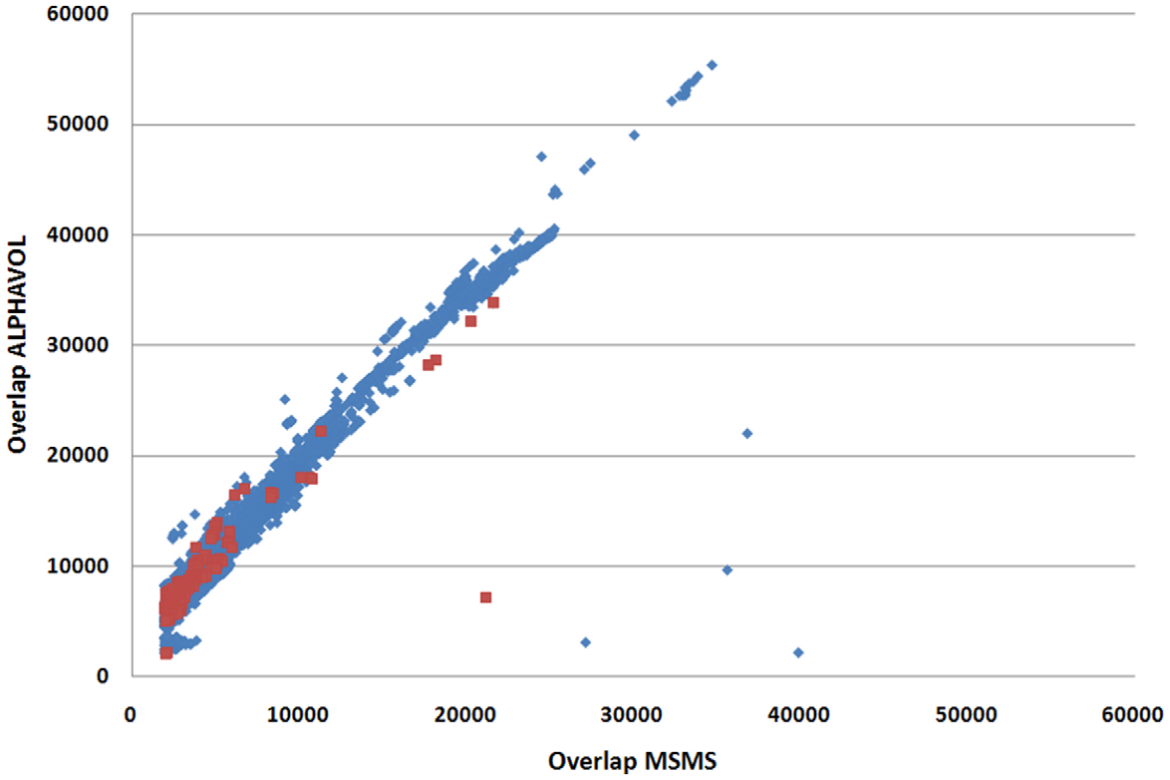
Correlation of the results generated by the two collision detection methods MSMS and ALPHAVOL. All overlap values detected by the two methods are shown in blue. The correlation of all results (12,772 protein interaction pairs) is 0.98. The filtered results are shown in red. Considering only these filtered results (4,874 protein interaction pairs), the correlation between the two methods slightly decreases to 0.90.

To quantify the extent of the collision between the two secondary proteins, we computed the volume of the overlap of the secondary proteins after superimposing the primary proteins. C_α_ atoms of the corresponding residues in the primary proteins were superimposed by a rigid-body transformation (translation and rotation) to minimize the RMSD between corresponding C_α_ atoms. The rotation was determined by Kearsley's quaternion method [17], posing the minimization as an eigenvalue problem, which is solved by a singular value decomposition. After optimal rigid-body superimposition of the primary proteins, the overlap volume of the secondary proteins was computed as the difference between the sum of the individual volumes of the secondary proteins and the volume of the union of the secondary proteins. For the computation of the molecular volumes, we calculated the solvent excluded volume with MSMS by Sanner *et al.* [18]. To confirm the results of this collision detection method, we alternatively computed the volume within the solvent accessible surface using ALPHAVOL [19]. Using these two complementary methods and measures, we filtered out few cases with numeric irregularities or instabilities. The high correlation of both methods (Figure 2) also confirms that both are suitable for the task of collision detection. We kept only those results in our dataset that were consistently identified by both collision detection methods.

## Results and Discussion

### Identification of Colliding Interaction Pairs

**Figure 3:**
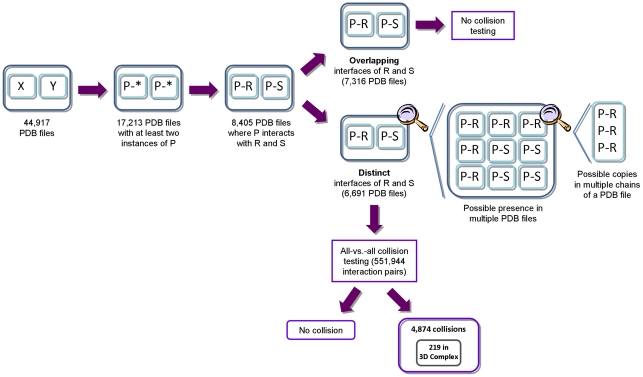
Overview and results of our structure collision approach. The flow chart illustrates the necessary steps for identifying 3D structure collisions of interacting proteins. Additionally, the number of PDB files and interaction pairs is given.

**Figure 4:**
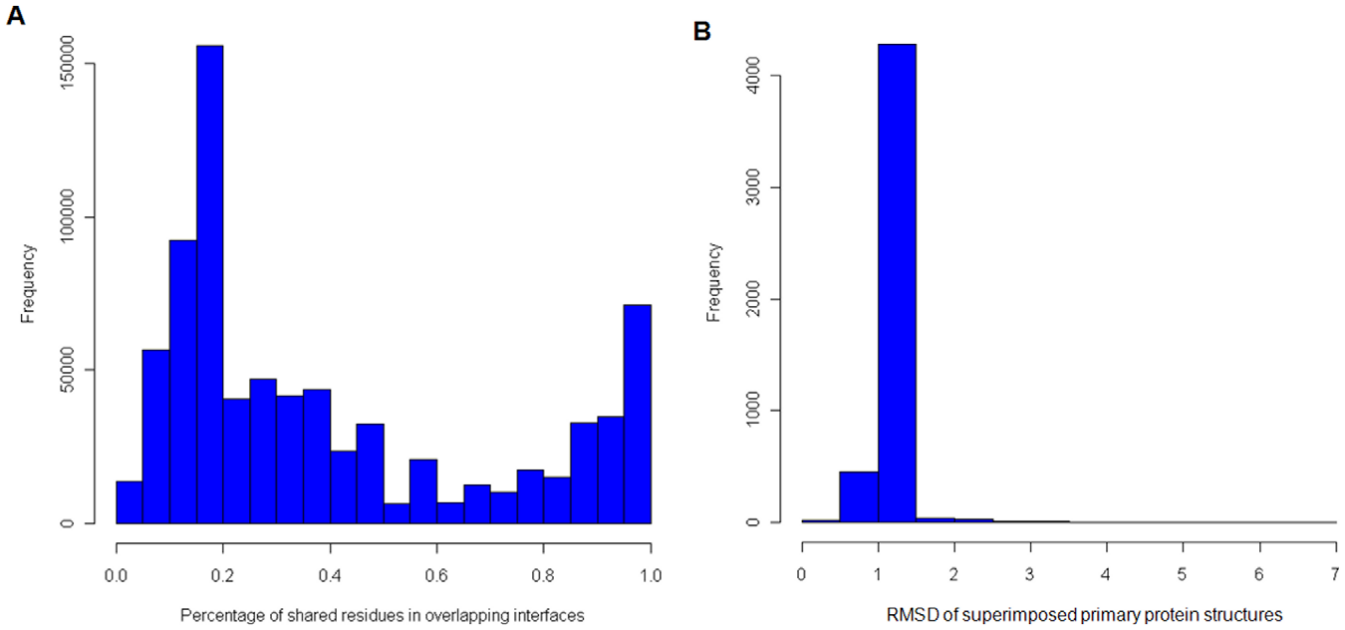
Histogram plots. (A) Percentage of shared residues in overlapping interfaces. (B) RMSD of superimposed primary protein structures.

**Figure 5:**
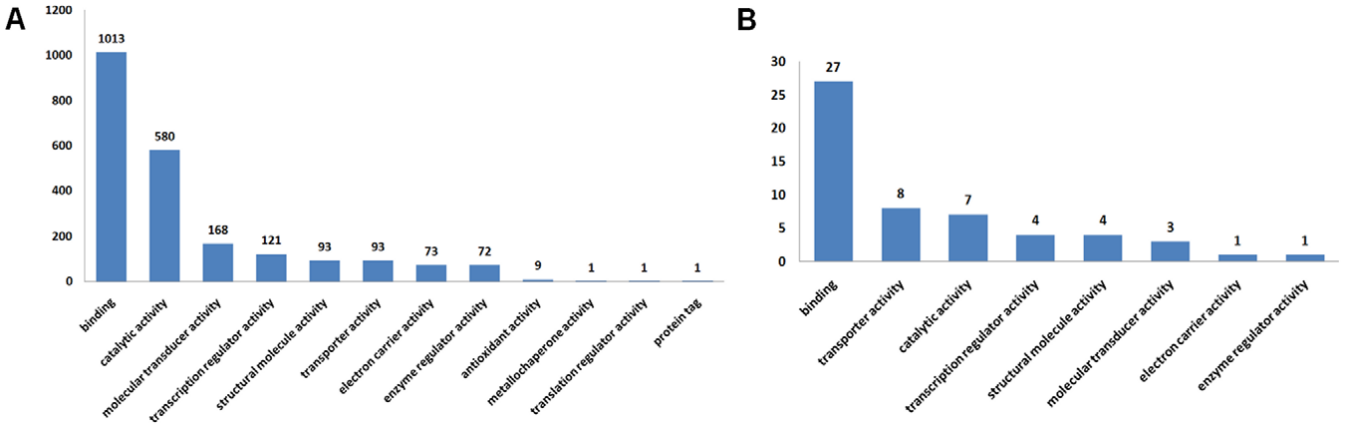
Functional Gene Ontology (GO) annotations of primary proteins. (A) GO molecular functions of the 1,432 primary proteins involved in the 551,994 interaction pairs that were tested for collisions. (B) GO molecular functions of the 37 primary proteins involved in the 4,874 interaction pairs with structural collisions.

The generation of the results proceeded in four main steps (see Figure 3). First, we identified all potential pairs of primary proteins, that is, all pairwise combinations of protein chains with identical UniProtKB accessions that were contained in at least two PDB structures and could serve as the primary proteins *P* of the interaction pair *P-R* and *P-S*. We found 4,832 proteins that were contained in at least two PDB files (out of a total of 17,213 relevant PDB files). This resulted in 1,145,086 possible combinations of potential primary proteins *P*. However, while the number of pairwise combinations of *P* is large, the number of involved proteins is much smaller. Many PDB files contain the same proteins, and one PDB file may contain multiple copies of the same protein. Thus the number of possible combinations of primary proteins grows quadratic. Second, to obtain the interaction pairs, we filtered for those primary proteins that interact with at least two different secondary proteins. When examining the primary proteins and their respective secondary proteins, we identified a total of 2,309,561 interaction pairs with different secondary proteins according to their UniProtKB accession numbers. Again, as above, the number of interaction pairs is much larger than the number of involved proteins because we need to combine all protein instances in an all-versus-all approach. Third, we compared the interface residues forming the interactions *P-R* and *P-S* in order to remove those interaction pairs with overlapping interfaces. Regarding the overlap of binding interfaces that were excluded due to our strict definition that requires no overlapping residues, most overlapping interfaces share at least 20% of the interface residues (average overlap 41%, Figure 4A). After this filtering step, 551,944 interaction pairs with distinct interfaces remained involving 1,432 primary proteins, which could be assigned to 6,691 PDB structures (see Figure 5A for the molecular functions of these proteins). Finally, all these interaction pairs were used as input for the collision detection method, and the volume overlap of the secondary proteins was computed for each interaction pair.

### Refinement of Collisions

We defined a collision to occur if both collision detection methods (MSMS and ALPHAVOL) consistently reported an overlap of the secondary proteins of at least 2000 Å^3^. Based on this definition, we identified 12,772 interaction pairs with colliding secondary proteins. As can be seen in Figure 2, the correlation of the overlap values produced by the two applied collision detection methods is 0.85, indicating a high reliability of the detected overlaps. The results were further refined and collisions were retained only if the RMSD of the superposition of the primary proteins was less than 7 Å, to avoid false positives due to improper superposition. For the large majority of the detected collisions, the RMSD was close to 1 Å, which is indicative of only small structural differences between the superimposed primary proteins (Figure 4B). We also excluded results where the sequence lengths of the primary proteins differ by more than 15 residues in order to avoid large structural differences between the primary proteins. Additionally, we required the alignment of the two primary proteins to cover at least thirty amino acids in order to remove interaction pairs where the primary proteins corresponded to small fragments of a full-length protein.

**Figure 6:**
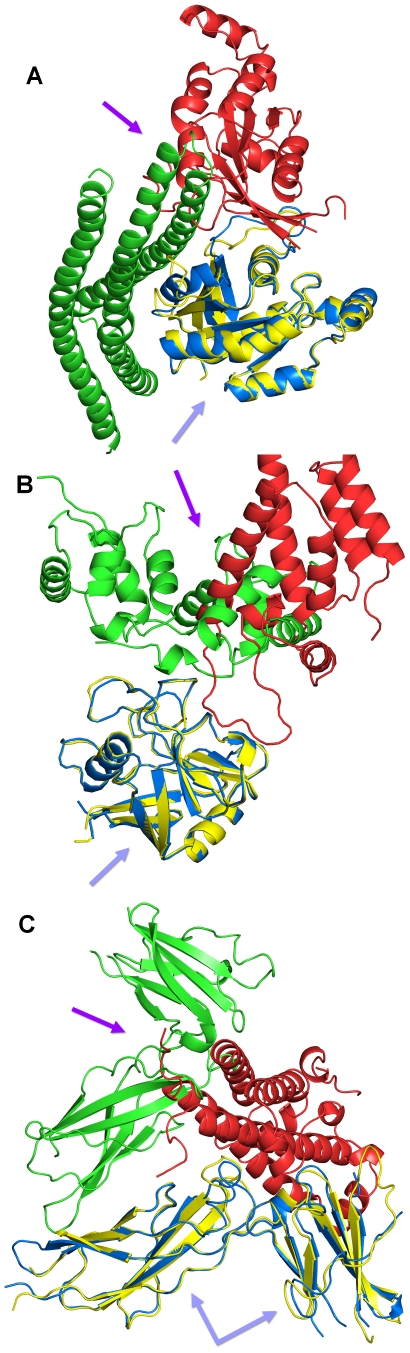
Visualization of three collision examples in cartoon representation. The structures of the primary proteins were superimposed (green arrows), and colliding regions are marked by red arrows. (A) Collision of the secondary proteins Arfaptin (PDB 1i4d, chain A, blue) and Rac1-GDP (PDB 2p2l, chain B, yellow), using Rac1-GDP as primary protein (PDB 1i4d, chain D, and PDB 2p2l, chain C). (B) Collision of calcineurin B subunit isoform 1 (PDB 1mf8, chain B, blue) and HIV-1 capsid protein (PDB 1m9x, chain D, yellow), using cyclophilin A as primary protein (PDB 1mf8, chain C, and PDB 1m9x, chain A). (C) Collisions of growth hormone receptor (PDB 1hwg, chain B, blue) and growth hormone (PDB 1a22, chain A, yellow), using soluble growth hormone receptor as primary protein (PDB 1hwg, chain C, and PDB 1a22, chain B).

These constraints reduced the number of colliding interaction pairs to 4,874 with an average RMSD of 1.23 Å and average overlap results of 2659 Å^3^ (MSMS) and 7049 Å^3^ (ALPHAVOL). The results were derived from 244 PDB structures, and 37 different primary proteins as well as 86 different secondary proteins participated in the interactions (see Figure 5B for the molecular functions of the primary proteins). These numbers show that many collisions of interaction pairs involved the same proteins. However, the number of colliding interaction pairs varied substantially with respect to the recurrences of the identified primary proteins, ranging from 1 to 3,777 structural instances. We also observed that, in 98% of the 4,874 interaction pairs, both the primary and the secondary protein chains comprise single SCOP domains [20]. Therefore, almost all collisions occur between single structural units of the participating proteins. One of the exceptions is illustrated in Figure 6C, where the extracellular domain of the growth hormone receptor contains two SCOP domains and the collision involves both domains. Notably, 97% of the collisions were derived from human interactions (see Tables S1 and S2 for details on all colliding protein interaction pairs).

### Analysis of Binding Interfaces

Since protein interactions are often formed by domain-domain interactions, we studied the binding interfaces of the detected interaction pairs in more detail. To this end, we analyzed Pfam-A domains [21] because their interactions are available in domain interaction databases. Our analysis revealed that, for most of our results (4,807 colliding interaction pairs, ∼98%), the interface residues of the primary proteins could not be exclusively assigned to a single Pfam-A domain-coding region. Instead, the interface residues belonged either to unstructured protein parts shared between one domain and additional unstructured parts of the primary proteins or shared between more than one domain and unstructured parts. This is particularly interesting since binding residues outside domain regions can stabilize the interaction additionally, but are not considered in domain interaction databases.

In the collision results, we found only 42 interaction pairs consisting of *P-R* and *P-S* where the interface residues of both primary proteins *P* could exclusively be assigned to the same single domain-coding region. The latter regions included 9 different Pfam-A domain families occurring in up to 13 interaction pairs, of which 5 domain families participate in catalytic activities (see Tables S3 and S4 for details).

### Filtering for Biological Interactions

To identify protein interactions that are reported as truly interacting, we used the database 3D Complex [22]. We kept only those results in which both protein interactions *P-R* and *P-S* are contained in 3D Complex. This reduced the number of colliding interaction pairs to 219. Of those, 5 collisions included multi-domain secondary proteins containing two SCOP domains, but the collisions always occurred in the binding domain. Most of the biological interaction pairs, i.e., 184, involved interactions between hemoglobin protein chains. For the other colliding interaction pairs, the number of instances was below ten. The over-representation of hemoglobin likely results from a bias in available PDB protein structures towards certain well-studied protein complexes (see Table S5 for a list of all 219 colliding interaction pairs and their instances). A manual investigation, however, revealed that all of the detected collisions occur as a consequence of non-natural structural conformations due to artificially constructed protein interactions.

### Examples of Structure Collisions

In the following, we show three examples of colliding protein interaction pairs (Figure 6). Figure 6A shows the superposition of Rac1 protein chains (primary protein) that are in complex with an Arfaptin fragment or crystallized as a Rac1 trimer (secondary proteins). Regarding the superposition of the Rac1 protein chains, 177 residues were aligned and the RMSD of the superimposed primary proteins is 1.99 Å. The overlap between the secondary proteins is ∼2215 Å^3^ according to MSMS and ∼5368 Å^3^ according to ALPHAVOL. Rac1 is a hub protein that forms part of more than 70 complexes in the PDB and participates in well over 200 different pairwise protein interactions (see BioMyn database at http://www.biomyn.de [23]). Arfaptin functions as an effector of Rac1 [24]. One chain of the Rac1 trimer collides with the Arfaptin fragment. Rac1 trimerisation was experimentally triggered by unnatural high levels of zinc that do not occur in living cells [25]. Therefore, this trimer complex is not expected to exist *in vivo*.

Figure 6B visualizes the superposition of the primary proteins cyclophilin A, which are in complex with a mutated HIV-1 capsid protein in one PDB structure and with a calcineurin B subunit in the other structure. 164 of the residues of the cyclophilin A chains could be aligned, resulting in a very precise superposition with an RMSD of 0.61 Å. The detected collision is larger than in the previous example, with ∼2807 Å^3^ reported by MSMS and ∼5995 Å^3^ by ALPHAVOL. Cyclophilins are enzymes involved in diverse functions including protein folding, transport and signaling [26]. They possess both sequence-specific binding and proline *cis*-*trans* isomerase activities. Cyclophilin A binds the HIV-1 capsid protein and facilitates virus replication. Calcineurin B participates in signaling for T-cell activation. The interaction between cyclophilin A and calcineurin B is part of a ternary complex with the immunosuppressive drug cyclosporin A. The latter binds to cyclophilin A, enabling both the binding and the inhibition of calcineurin B and is thus an artificial construct [27].

Figure 6C shows a collision between a growth hormone receptor (GHR) and a growth hormone (GH), which are both crystallized in interaction with the primary protein GHR. GHR was aligned with an RMSD of 1.65 Å ranging over 186 residues. A collision was detected between the second GHR from the dimer with the GH chain from the monomer, and MSMS reported ∼2330 Å^3^ and ALPHAVOL ∼6194 Å^3^. The active signaling complex has a stoichiometry of one GH molecule bound to two copies of its receptor [28]. The detected collision originates from the artificial construct of a GHR monomer in complex with GH (PDB 1a22), which does not exist *in vivo* [29].

### Conclusions

Our structure collision approach enabled the discovery of several cases of protein interaction pairs with colliding protein structures. We did not detect biologically relevant 3D collisions of simultaneously possible protein interactions, but our analysis was limited by the low number of structurally determined protein complexes in the PDB. The identified collisions usually occurred between protein structures that were determined under different experimental conditions to study alternative conformations or quaternary structures of the proteins. Nevertheless, our analysis approach revealed several interesting occurrences of structural collisions.

Therefore, it is still important for future studies of protein interaction networks that separate binding interfaces might not imply simultaneously possible protein interactions. The functional implications of spatially colliding interaction partners can be manifold and similar to those of overlapping or identical binding sites such as the temporal control or inhibition of protein binding. In particular, structure collisions might be due to disease-associated mutations or constitute essential regulation mechanisms for transient protein interactions as they occur in signaling processes [30]. Here, collisions might involve adaptor and scaffold proteins and their interaction partners. These proteins frequently have a greater number of interaction partners than binding interfaces [23]. Thus, the combination of proteins that bind simultaneously to another protein at a specific time point or cellular location needs to be well-defined [31]. Regulatory mechanisms different from the number of binding interfaces are needed for understanding the binding of specific combinations of proteins.

Finally, aside from the lack of structural data, there might be other reasons for not observing biologically relevant collisions in our study. For instance, PDB structures often consist of single protein domains as independently folded structural units instead of complete proteins. Therefore, different domains from a multi-domain protein can be found in multiple PDB structure chains. Modeling structural linkers between the domains is still a very difficult task and cannot be performed at large scale yet. Consequently, we might have missed collisions between protein chains that bind the same protein in separate domains. Further issues are the existence of disordered regions and allosteric effects [32], [33], i.e., the flexible nature of proteins, which might promote or prevent collisions. However, the required flexibility data on minor and major structural movements have not been available yet for such large-scale analyses as performed by us as well as other researchers. When more comprehensive structural datasets of protein complexes will be available, further work might shed light on the presence and functional relevance of naturally occurring structure collisions.

## Supporting Information

Table S1 **List of colliding protein interaction pairs.**
(PDF)

Table S2 **GO annotations of proteins.**
(PDF)

Table S3 **Protein interactions with single-domain interface.**
(PDF)

Table S4 **GO annotations of Pfam domains.**
(PDF)

Table S5 **List of colliding protein interaction pairs after filtering with 3D Complex.**
(PDF)
