# Supplementary material for: Structure Collisions between Interacting Proteins
Source: PLoS One. 2011 Jun 2;6(6):e19581. doi: 10.1371/journal.pone.0019581 (PMC3107212; doi:10.1371/journal.pone.0019581)
Supplement: Table S1 — List of colliding protein interaction pairs. (PDF) [file pone.0019581.s001.pdf]

Table S1. List of colliding protein interaction pairs. For each interaction pair, the gene names and species of the primary and secondary proteins are listed together with the number of instances within the 4,874 results. The primary proteins are shown in the first column, the secondary proteins in the fourth column, and the number of instances in the last column.

Primary Protein	Protein Name	Species	Secondary Proteins	Protein Names	Species of Secondary Proteins	Instances
P69905	Hemoglobin subunit alpha	H.sapiens	[P69905, P68871]	[Hemoglobin subunit alpha, Hemoglobin subunit beta]	[H.sapiens, H.sapiens]	3,777
P69905	Hemoglobin subunit alpha	H.sapiens	[P69905, P02042]	[Hemoglobin subunit alpha, Hemoglobin subunit delta]	[H.sapiens, H.sapiens]	756
P69905	Hemoglobin subunit alpha	H.sapiens	[P02100, P69905]	[Hemoglobin subunit epsilon, Hemoglobin subunit alpha]	[H.sapiens, H.sapiens]	151
P00459	Nitrogenase iron protein 1	A.vinelandii	[P07328, P07329]	[Nitrogenase molybdenum-iron protein alpha chain, Nitrogenase molybdenum-iron protein beta chain]	[A.vinelandii, A.vinelandii]	13
P01901	H-2 class I histocompatibility antigen, K-B alpha chain	M.musculus	[P01901, P01731]	[H-2 class I histocompatibility antigen, K-B alpha chain, T-cell surface glycoprotein CD8 alpha chain]	[M.musculus, M.musculus]	12
P62560	Exotoxin type A	S.pyogenes	[P62560, P01851]	[Exotoxin type A, T-cell receptor beta-2 chain C region]	[S.pyogenes, M.musculus]	12
P68135	Actin, alpha skeletal muscle	O.cuniculus	[P13538, P68135]	[Myosin heavy chain, skeletal muscle, adult, Actin, alpha skeletal muscle]	[G.gallus, O.cuniculus]	11
P01903	HLA class II histocompatibility antigen, DR alpha chain	H.sapiens	[P04229, Q30154]	[HLA class II histocompatibility antigen, DRB1-1 beta chain, HLA class II histocompatibility antigen, DRB5 beta chain]	[H.sapiens, H.sapiens]	10
P02881	Monellin chain A	D.cumminsii	[P02881, P02882]	[Monellin chain A, Monellin chain B]	[D.cumminsii, D.cumminsii]	8
P04229	HLA class II histocompatibility antigen, DRB1-1 beta chain	H.sapiens	[P01903, P01848]	[HLA class II histocompatibility antigen, DR alpha chain, T-cell receptor alpha chain C region]	[H.sapiens, H.sapiens]	8
P32851	Syntaxin-1A	R.norvegicus	[P60881, P32851]	[Synaptosomal-associated protein 25, Syntaxin-1A]	[R.norvegicus, R.norvegicus]	7
P01903	HLA class II histocompatibility antigen, DR alpha chain	H.sapiens	[Q30154, P01892]	[HLA class II histocompatibility antigen, DRB5 beta chain, HLA class I histocompatibility antigen, A-2 alpha chain]	[H.sapiens, H.sapiens]	6
P60881	Synaptosomal-associated protein 25	R.norvegicus	[P60881, P32851]	[Synaptosomal-associated protein 25, Syntaxin-1A]	[R.norvegicus, R.norvegicus]	6
P10912	Growth hormone receptor	H.sapiens	[P01241, P10912]	[Somatotropin, Growth hormone receptor]	[H.sapiens, H.sapiens]	5
P01903	HLA class II histocompatibility antigen, DR alpha chain	H.sapiens	[Q30126, Q30154]	[HLA class II histocompatibility antigen, DRB5 beta chain, HLA class II histocompatibility antigen, DRB5 beta chain]	[H.sapiens, H.sapiens]	4
P58154	Acetylcholine-binding protein	L.stagnalis	[P01391, P58154]	[Long neurotoxin 1, Acetylcholine-binding protein]	[N.kaouthia, L.stagnalis]	4
P68871	Hemoglobin subunit beta	H.sapiens	[P69905, P68871]	[Hemoglobin subunit alpha, Hemoglobin subunit beta]	[H.sapiens, H.sapiens]	4
P04229	HLA class II histocompatibility antigen, DRB1-1 beta chain	H.sapiens	[P04229, P01848]	[HLA class II histocompatibility antigen, DRB1-1 beta chain, T-cell receptor alpha chain C region]	[H.sapiens, H.sapiens]	4

P13272	Cytochrome b-c1 complex subunit Rieske, mitochondrial	B.taurus	[P00157, P13271]	[Cytochrome b, Cytochrome b-c1 complex subunit 8]	[B.taurus, B.taurus]	4
P32851	Syntaxin-1A	R.norvegicus	[Q9GM34, P32851]	[Synaptosomal-associated protein, Syntaxin-1A]	[M.fascicularis, R.norvegicus]	4
P12943	Periplasmic [NiFe] hydrogenase small subunit	D.gigas	[P12944, P12943]	[Periplasmic [NiFe] hydrogenase large subunit, Periplasmic [NiFe] hydrogenase small subunit]	[D.gigas, D.gigas]	4
P63000	Ras-related C3 botulinum toxin substrate 1	H.sapiens	[P53365, P63000]	[Arfaptin-2, Ras-related C3 botulinum toxin substrate 1]	[H.sapiens, H.sapiens]	4
P62937	Peptidyl-prolyl cis-trans isomerase A	H.sapiens	[P63098, Q4JL05]	[Calcineurin subunit B type 1, Gag polyprotein]	[H.sapiens, HIV-1]	3
P62937	Peptidyl-prolyl cis-trans isomerase A	H.sapiens	[P63098, Q4JL04]	[Calcineurin subunit B type 1, Gag polyprotein]	[H.sapiens, HIV-1]	3
P08160	Early 35 kDa protein	A.californica	[P08160, Q14790]	[Early 35 kDa protein, Caspase-8]	[A.californica, H.sapiens]	3
Q06851	Cellulosomal-scaffolding protein A	C.thermocellum	[Q06851, P51584]	[Cellulosomal-scaffolding protein A, Endo-1, 4-beta-xylanase Y]	[C.thermocellum, C.thermocellum]	3
P68390	Ovomucoid	M.gallopavo	[P00761, P00766]	[Trypsin, Chymotrypsinogen A]	[S.scrofa, B.taurus]	3
P01731	T-cell surface glycoprotein CD8 alpha chain	M.musculus	[P01887, P01731]	[Beta-2-microglobulin, T-cell surface glycoprotein CD8 alpha chain]	[M.musculus, M.musculus]	3
Q52L64	ENSMUSG00000076577 protein	M.musculus	[P01868, P06213]	[Ig gamma-1 chain C region secreted form, Insulin receptor]	[M.musculus, H.sapiens]	2
P62937	Peptidyl-prolyl cis-trans isomerase A	H.sapiens	[P63098, P62937]	[Calcineurin subunit B type 1, Peptidyl-prolyl cis-trans isomerase A]	[H.sapiens, H.sapiens]	2
P61889	Malate dehydrogenase	E.coli	[A1AGC9, P61889]	[Malate dehydrogenase, Malate dehydrogenase]	[E.coli, E.coli]	2
Q9H1K0	Rabenosyn-5	H.sapiens	[P20338, P35285]	[Ras-related protein Rab-4A, Ras-related protein Rab-22A]	[H.sapiens, M.musculus]	2
P01903	HLA class II histocompatibility antigen, DR alpha chain	H.sapiens	[Q48898, P01850]	[Superantigen, T-cell receptor beta chain C region]	[M.arthritidis, H.sapiens]	2
P01903	HLA class II histocompatibility antigen, DR alpha chain	H.sapiens	[P01911, Q30154]	[HLA class II histocompatibility antigen, DRB1-15 beta chain, HLA class II histocompatibility antigen, DRB5 beta chain]	[H.sapiens, H.sapiens]	2
P04275	von Willebrand factor	H.sapiens	[Q7LZK8, P04275]	[Bitiscetin subunit beta, von Willebrand factor]	[B.arietans, H.sapiens]	2
O07347	Signal recognition particle protein	T.aquaticus	[P83749, O07347]	[Cell division protein ftsY, Signal recognition particle protein]	[T.aquaticus, T.aquaticus]	2
P02787	Serotransferrin	H.sapiens	[P02786, P02787]	[Transferrin receptor protein 1, Serotransferrin]	[H.sapiens, H.sapiens]	2
P01837	Ig kappa chain C region	M.musculus	[P14013, P13726]	[Outer surface protein A, Tissue factor]	[B.burgdorferi, H.sapiens]	2
P03680	DNA polymerase	B.phage	[P03681, P03680]	[DNA terminal protein, DNA polymerase]	[B.phage, B.phage]	2
P27152	30S ribosomal protein S5	T.thermophilus	[P24319, Q5SHQ2]	[30S ribosomal protein S8, 30S ribosomal protein S8]	[T.thermophilus, T.thermophilus]	2
P01966	Hemoglobin subunit alpha	B.taurus	[P02070, P01966]	[Hemoglobin subunit beta, Hemoglobin subunit alpha]	[B.taurus, B.taurus]	2

P62937	Peptidyl-prolyl cis-trans isomerase A	H.sapiens	[P63100, P62937]	[Calcineurin subunit B type 1, Peptidyl-prolyl cis-trans isomerase A]	[R.norvegicus, H.sapiens]	1
P62937	Peptidyl-prolyl cis-trans isomerase A	H.sapiens	[P63098, Q8Q0Z0]	[Calcineurin subunit B type 1, Gag polyprotein]	[H.sapiens, HIV-1]	1
P01834	Ig kappa chain C region	H.sapiens	[P01834, P01857]	[Ig kappa chain C region, Ig gamma-1 chain C region]	[H.sapiens, H.sapiens]	1
Q6GMX8	IGKC protein	H.sapiens	[Q6GMX8, P15692]	[IGKC protein, Vascular endothelial growth factor A]	[H.sapiens, H.sapiens]	1
P63208	S-phase kinase-associated protein 1	H.sapiens	[Q9Y297, Q13309]	[F-box/WD repeat-containing protein 1A, S-phase kinase-associated protein 2]	[H.sapiens, H.sapiens]	1
P60881	Synaptosomal-associated protein 25	R.norvegicus	[P63027, P60881]	[Vesicle-associated membrane protein 2, Synaptosomal-associated protein 25]	[H.sapiens, R.norvegicus]	1
P60881	Synaptosomal-associated protein 25	R.norvegicus	[P60881, Q9N0Y0]	[Synaptosomal-associated protein 25, Vesicle-associated membrane protein 2]	[R.norvegicus, M.mulatta]	1
P60881	Synaptosomal-associated protein 25	R.norvegicus	[P60881, P63045]	[Synaptosomal-associated protein 25, Vesicle-associated membrane protein 2]	[R.norvegicus, R.norvegicus]	1
P60881	Synaptosomal-associated protein 25	R.norvegicus	[P63027, P32851]	[Vesicle-associated membrane protein 2, Syntaxin-1A]	[H.sapiens, R.norvegicus]	1
P60881	Synaptosomal-associated protein 25	R.norvegicus	[Q9N0Y0, P32851]	[Vesicle-associated membrane protein 2, Syntaxin-1A]	[M.mulatta, R.norvegicus]	1
P60881	Synaptosomal-associated protein 25	R.norvegicus	[P63045, P32851]	[Vesicle-associated membrane protein 2, Syntaxin-1A]	[R.norvegicus, R.norvegicus]	1
P62988	Ubiquitin	H.sapiens	[Q45TR8, P62988]	[Ubiquitin, Ubiquitin]	[M.domestica, H.sapiens]	1
P12497	Gag-Pol polyprotein	HIV-1	[Q9JL77, P12497]	[Anti-myosin immunoglobulin heavy chain variable region, Gag-Pol polyprotein]	[M.musculus, HIV-1]	1
P0AEX9	Maltose-binding periplasmic protein	E.coli	[Q9VUQ5, P14120]	[Protein argonaute-2, 60S ribosomal protein L30]	[D.melanogaster, S.cerevisiae]	1
P68390	Ovomucoid	M.gallopavo	[P00761, P08246]	[Trypsin, Leukocyte elastase]	[S.scrofa, H.sapiens]	1
P63000	Ras-related C3 botulinum toxin substrate 1	H.sapiens	[Q6RUV5, P53365]	[Ras-related C3 botulinum toxin substrate 1, Arfaptin-2]	[R.norvegicus, H.sapiens]	1
