# Supplementary material for: Structure Collisions between Interacting Proteins
Source: PLoS One. 2011 Jun 2;6(6):e19581. doi: 10.1371/journal.pone.0019581 (PMC3107212; doi:10.1371/journal.pone.0019581)
Supplement: Table S2 — GO annotations of proteins. (PDF) [file pone.0019581.s002.pdf]

Table S2. GO annotations of proteins. Primary and secondary proteins contained in the 4,874 protein interaction pairs are listed together with their GO annotations.

Protein	Biological Process	Molecular Function	Cellular Component
P01901	antigen processing and presentation of ...; defense response to bacterium; immune response; positive regulation of T cell mediated cytotoxicity	peptide antigen binding	external side of plasma membrane; integral to membrane; MHC class I protein complex
P01731	cell surface receptor linked signaling pathway; cytotoxic T cell differentiation; positive regulation of calcium-mediated signaling; T cell mediated immunity	protein homodimerization activity	external side of plasma membrane; integral to membrane
P69905	oxygen transport	heme binding; oxygen binding; oxygen transporter activity; protein binding	hemoglobin complex
P68871	nitric oxide transport; oxygen transport; positive regulation of nitric oxide biosynthetic process; regulation of blood pressure & blood vessel size	heme binding; hemoglobin binding; oxygen binding; oxygen transporter activity	hemoglobin complex
P02042	oxygen transport	heme binding; oxygen binding; oxygen transporter activity	hemoglobin complex
P02100	oxygen transport	heme binding; oxygen binding; oxygen transporter activity	hemoglobin complex
Q52L64			
P01868	antibacterial humoral response; antibody-dependent cellular cytotoxicity; complement activation, classical pathway; phagocytosis, engulfment; phagocytosis, recognition; positive regulation of phagocytosis; positive regulation of type I & IIa hypersensitivity	antigen binding	immunoglobulin complex, circulating
P06213	activation of MAPK & protein kinase B activity; carbohydrate metabolic process; G-protein coupled receptor protein signaling pathway; glucose homeostasis; heart morphogenesis; peptidyl-tyrosine phosphorylation; positive regulation of …; protein autophosphorylation; protein heterotetramerization; regulation of embryonic development & gene-specific transcription; transformation of host cell by virus	ATP binding; GTP binding; insulin binding; insulin receptor activity; insulin receptor substrate binding; insulin-like growth factor I binding; insulin-like growth factor II binding; insulin-like growth factor receptor binding; metal ion binding; phosphoinositide 3-kinase binding; PTB domain binding; receptor signaling protein tyrosine kinase activity; SH2 domain binding	caveola; endosome membrane; insulin receptor complex; microsome
P62937	initiation of viral infection; interspecies interaction between organisms; protein folding; provirus integration; regulation of viral genome replication	peptide binding; peptidyl-prolyl cis-trans isomerase activity; unfolded protein binding; virion binding	cytosol; extracellular region; nucleus
P63098	activation of pro-apoptotic gene products	calcium ion binding; calcium-dependent protein serine/threonine phosphatase activity; calmodulin binding	calcineurin complex; cytosol
P63100	protein dephosphorylation	calcium ion binding; phosphoprotein phosphatase activity; protein binding	
Q4JL05	viral reproduction		
Q4JL04	viral reproduction		
Q8Q0Z0	viral reproduction	RNA binding; structural molecule activity; zinc ion binding	cytoplasm; host cell cytoplasm; host cell nucleus; nucleus; viral capsid
P00459	nitrogen fixation; oxidation reduction	ATP binding; 4 iron, 4 sulfur cluster binding; metal ion binding; nitrogenase activity	molybdenum-iron nitrogenase complex
P07328	nitrogen fixation; oxidation reduction	ATP binding; iron-sulfur cluster binding; metal ion binding; nitrogenase activity	molybdenum-iron nitrogenase complex
P07329	nitrogen fixation; oxidation reduction	ATP binding; iron-sulfur cluster binding; metal ion binding; nitrogenase activity	molybdenum-iron nitrogenase complex
P61889	anaerobic respiration; fermentation; glycolysis; malate metabolic process; oxidation reduction; tricarboxylic acid cycle	binding; L-malate dehydrogenase activity	cytosol; membrane; peripheral to membrane of membrane fraction
A1AGC9	glycolysis; malate metabolic process; oxidation reduction; tricarboxylic acid cycle	binding; L-malate dehydrogenase activity	
P01834	immune response	antigen binding; protein binding	extracellular region
P01857	immune response	antigen binding; protein binding	extracellular region; membrane fraction

P62560	pathogenesis		extracellular region
P01851		receptor activity	integral to membrane
P02881			
P02882			
Q9H1K0	endosome transport; protein transport	protein binding; zinc ion binding	early endosome & plasma membrane
P20338	small GTPase mediated signal transduction	GDP binding; GTP binding; GTPase activity	cytoplasm; membrane
P35285	protein transport; small GTPase mediated signal transduction	GTP binding; GTPase activity; protein binding	endosome & plasma membrane
P01903	antigen processing and presentation of …; immune response	MHC class II receptor activity	ER membrane; Golgi apparatus; integral to plasma membrane; late endosome & lysosomal membrane; MHC class II protein complex
Q48898			
P01850	immune response	protein binding; receptor activity	integral to membrane; plasma membrane
P04229	antigen processing and presentation of …; immune response		ER membrane; Golgi apparatus; integral to plasma membrane; late endosome & lysosomal membrane; MHC class II protein complex
Q30154	antigen processing and presentation of …; immune response		ER membrane; Golgi apparatus; integral to plasma membrane; late endosome & lysosomal membrane; MHC class II protein complex
P01892	antigen processing and presentation of …; immune response; interspecies interaction between organisms	protein binding	early endosome membrane; Golgi membrane; integral to membrane; MHC class I protein complex
P01911	antigen processing and presentation of …; immune response		ER membrane; Golgi apparatus; integral to plasma membrane; late endosome & lysosomal membrane; MHC class II protein complex
Q30126	antigen processing and presentation of …; immune response		ER membrane; Golgi apparatus; integral to plasma membrane; late endosome & lysosomal membrane; MHC class II protein complex
Q6GMX8			
P15692	basophil chemotaxis; cellular response to hypoxia; induction of positive chemotaxis; platelet-derived growth factor receptor signaling pathway; positive regulation of ...; regulation of cell shape; vasculogenesis	cell surface binding; chemoattractant activity; cytokine activity; extracellular matrix binding; fibronectin binding; growth factor activity; heparin binding; platelet-derived growth factor receptor binding; protein heterodimerization & homodimerization activity; vascular endothelial growth factor receptor binding	cell surface; extracellular space; membrane; stored secretory granule
P58154	ion transport	extracellular ligand-gated ion channel activity; receptor activity	cell junction; extracellular region; integral to membrane; postsynaptic membrane
P01391	pathogenesis; synaptic transmission	acetylcholine receptor inhibitor activity	extracellular region; postsynaptic membrane
P08160	anti-apoptosis; apoptosis	caspase inhibitor activity; protein binding	
Q14790	activation of pro-apoptotic gene products; induction of apoptosis by extracellular signals; positive regulation of I-kappaB kinase/NF-kappaB cascade; proteolysis involved in …; response to tumor necrosis factor	cysteine-type endopeptidase activity; identical protein binding; protein binding	cytoskeleton; cytosol; death-inducing signaling complex; mitochondrial outer membrane
P04275	cell-substrate adhesion; platelet activation; protein homooligomerization	chaperone, collagen, glycoprotein, immunoglobulin, integrin & protease binding; protein homodimerization activity; protein N-terminus binding	ER; platelet alpha granule; proteinaceous extracellular matrix; Weibel-Palade body
Q7LZK8	pathogenesis	sugar binding	extracellular region
P63208	positive regulation of ubiquitin-protein ligase activity involved in …; SCF-dependent proteasomal ubiquitin-dependent protein catabolic process	protein binding; ubiquitin-protein ligase activity	cytosol; nucleoplasm; SCF ubiquitin ligase complex

Q9Y297	interspecies interaction between organisms; positive regulation of ubiquitin-protein ligase activity involved in …; signal transduction; ubiquitin-dependent protein catabolic process; Wnt receptor signaling pathway	ligase activity; protein binding	cytosol; nucleus
Q13309	cell proliferation; G1/S transition of mitotic cell cycle	protein binding	SCF ubiquitin ligase complex
O07347	SRP-dependent cotranslational protein targeting to membrane	7S RNA binding; GTP binding; nucleoside-triphosphatase activity; protein binding	signal recognition particle, ER targeting
P83749	cell cycle; cell division; SRP-dependent cotranslational protein targeting to membrane	GTP binding; nucleoside-triphosphatase activity; protein binding; RNA binding	plasma membrane; ribonucleoprotein complex
P02787	cellular iron ion homeostasis; iron ion transport	ferric iron binding	apical & basal plasma membrane; coated pit; early & late & recycling endosome; endocytic vesicle; extracellular region; perinuclear region of cytoplasm; stored secretory granule
P02786	cellular iron ion homeostasis; endocytosis; interspecies interaction between organisms; proteolysis	peptidase activity; transferrin receptor activity	coated pit; endosome; integral to plasma membrane; melanosome
P60881	axonogenesis; calcium ion-dependent exocytosis of neurotransmitter; endosome transport; exocytosis; growth hormone secretion; long-term memory; positive regulation of insulin secretion; regulation of synaptogenesis; sleep	SNAP receptor activity; myosin binding; protein N-terminus binding; protein domain specific binding; syntaxin-1 binding; voltage-gated potassium channel activity	cell junction; endosome; growth cone; membrane raft; perinuclear region of cytoplasm; SNARE complex; synapse; synaptosome; voltage-gated potassium channel complex
P32851	intracellular protein transport; positive regulation of …; response to gravity; synaptic vesicle docking involved in exocytosis	ATP-dependent & calcium-dependent protein binding; glycoprotein & myosin head/neck binding; protein N-terminus binding; protein binding, bridging; protein domain specific binding; protein heterodimerization activity; SNAP receptor activity; SNARE binding;	actomyosin; cell junction; integral to membrane; stored secretory granule; synaptic vesicle membrane; SNARE complex; synaptosome
P63027	cellular membrane organization; post-Golgi vesicle-mediated transport	protein binding	cell junction; clathrin sculpted gamma-aminobutyric acid & glutamate transport vesicle membrane; integral to plasma membrane; secretory granule membrane; synaptic vesicle membrane; synaptosome
Q9N0Y0	vesicle-mediated transport		cell junction; integral to membrane; synaptic vesicle membrane; synaptosome
P63045	protein complex assembly; protein transport; regulation of exocytosis; response to glucose stimulus; vesicle-mediated transport	myosin binding; protein C-terminus binding; protein complex binding; syntaxin-1 binding	cell junction; integral to membrane; microsome; stored secretory granule; synaptic vesicle membrane; SNARE complex; synaptosome
P62988	anaphase-promoting complex-dependent proteasomal ubiquitin-dependent protein catabolic process; anti-apoptosis; apoptosis; axon guidance; ER-associated protein catabolic process; induction of apoptosis by …; long-term strengthening of neuromuscular junction; negative & positive regulation of ubiquitin-protein ligase activity involved in …; positive regulation of transcription; protein ubiquitination; regulation of synaptic plasticity	protein binding; transcription regulator activity	endosome membrane; nucleoplasm
Q45TR8			
P01848	cellular defense response	MHC protein binding; peptide antigen binding; receptor activity	integral to plasma membrane
Q06851	cell wall organization; cellulose catabolic process	carbohydrate binding; hydrolase activity, hydrolyzing …; protein binding	extracellular region; peptidoglycan-based cell wall
P51584	xylan catabolic process	cation binding; endo-1,4-beta-xylanase activity; protein binding	
P01837		antigen binding	
P14013			cell outer membrane; lipid-anchor
P13726	activation of blood coagulation via …; activation of caspase activity; activation of plasma proteins involved in …; anti-apoptosis; blood coagulation, extrinsic pathway, positive regulation of …	cell surface binding; phospholipid binding; protease binding	extracellular matrix; extracellular space; integral to membrane; intrinsic to external side of plasma membrane

P12497	initiation of viral infection; proteolysis; provirus integration; RNA-dependent DNA replication; transposition; viral procapsid maturation	aspartic-type endopeptidase activity; DNA & RNA binding; DNA-directed & RNA-directed DNA polymerase activity; integrase activity; ribonuclease H activity; structural molecule activity; zinc ion binding	cytosol; host cell cytoplasm; host cell nucleus; host cell plasma membrane; membrane; viral capsid
Q9JL77			
P03680	DNA replication	3'-5' exonuclease activity; DNA binding; DNA-directed DNA polymerase activity; nucleotide binding	
P03681	DNA replication, synthesis of RNA primer; DNA-protein covalent cross-linking		
P13272	electron transport chain; transport	2 iron, 2 sulfur cluster binding; metal ion binding; ubiquinol-cytochrome-c reductase activity	integral to membrane; mitochondrial inner membrane; respiratory chain
P00157	respiratory electron transport chain; transport	electron carrier activity; metal ion binding; oxidoreductase activity	integral to membrane; mitochondrial inner membrane; respiratory chain
P13271	electron transport chain	ubiquinol-cytochrome-c reductase activity	mitochondrial inner membrane; respiratory chain
P68135	skeletal muscle fiber development; skeletal muscle thin filament assembly	ATP binding	stress fiber; striated muscle thin filament
P13538		actin binding; ATP binding; calmodulin binding; motor activity	myofibril; myosin filament
Q9GM34			synaptosome
P0AEX9	maltodextrin transport	disaccharide binding; maltose transmembrane transporter activity	ABC transporter complex, substrate-binding …; periplasmic space
Q9VUQ5	defense response to virus; dsRNA transport; heterochromatin organization involved in chromatin silencing; interspecies interaction between organisms; negative regulation of viral genome replication; pole cell formation; production of siRNA involved in …; salivary gland cell autophagic cell death; segment polarity determination; siRNA loading onto RISC involved in …; syncytial nuclear migration; targeting of mRNA for destruction involved in RNA interference	endoribonuclease activity; protein binding; siRNA binding	cytoplasm; RISC-loading complex; RNA-induced silencing complex
P14120	negative regulation of nuclear mRNA splicing, via …; negative regulation of translation; rRNA processing; translation	pre-mRNA 5'-splice site binding; protein binding; structural constituent of ribosome	cytosolic large ribosomal subunit
P27152	translation	rRNA binding; structural constituent of ribosome	small ribosomal subunit
P24319	translation	rRNA binding; structural constituent of ribosome	ribosome
Q5SHQ2	translation	rRNA binding; structural constituent of ribosome	ribosome
P68390		serine-type endopeptidase inhibitor activity	extracellular region
P00761	digestion; proteolysis	metal ion binding; serine-type endopeptidase activity	extracellular region
P00766	digestion; proteolysis	serine-type endopeptidase activity	extracellular region
P08246	cellular calcium ion homeostasis; negative regulation of …; positive regulation of ...; protein catabolic process; proteolysis; response to UV	bacterial cell surface binding; cytokine binding; heparin binding	cell surface; extracellular region; stored secretory granule
P01966	oxygen transport	heme binding; oxygen binding; oxygen transporter activity; protein binding	hemoglobin complex
P02070	oxygen transport	heme binding; oxygen binding; oxygen transporter activity	hemoglobin complex
P12943	oxidation reduction	3 iron, 4 sulfur & 4 iron, 4 sulfur cluster binding; cytochrome-c3 hydrogenase activity; ferredoxin hydrogenase activity; metal ion binding; NADH dehydrogenase (ubiquinone) activity	ferredoxin hydrogenase complex; periplasmic space
P12944	oxidation reduction	cytochrome-c3 hydrogenase activity; ferredoxin hydrogenase activity; nickel ion binding	periplasmic space

P10912	2-oxoglutarate, allantoin, citrate, creatine & creatinine metabolic process; activation of JAK2 & MAPK kinase activity; endocytosis; fatty acid metabolic process; growth hormone receptor signaling pathway; insulin-like growth factor receptor signaling pathway; isoleucine metabolic process; multicellular organismal metabolic process; oxaloacetate metabolic process; positive regulation of …; receptor internalization; response to cycloheximide & estradiol stimulus; succinate, taurine & valine metabolic process	growth factor binding; peptide hormone binding; proline-rich region binding; protein homodimerization activity; protein kinase binding	cell surface; extracellular space; growth hormone receptor complex; integral to plasma membrane
P01241	glucose transport; growth hormone receptor signaling pathway; positive regulation of …; JAK-STAT cascade; response to estradiol stimulus	growth factor activity; growth hormone receptor binding; hormone activity; metal ion binding; prolactin receptor binding	extracellular space
P63000	actin filament polymerization; anatomical structure morphogenesis; apoptosis; cell-matrix adhesion; cellular component movement; induction of apoptosis by …; inflammatory response; lamellipodium assembly; localization within membrane; negative regulation of receptor-mediated endocytosis; positive regulation of Rho protein signal transduction & lamellipodium assembly; regulation of hydrogen peroxide metabolic process & respiratory burst; ruffle organization; small GTPase mediated signal transduction	GTP binding; GTP-dependent protein binding; GTPase activity; thioesterase binding	cytosol; melanosome; plasma membrane
P53365	actin cytoskeleton organization; cellular component movement; lamellipodium assembly; ruffle organization; small GTPase mediated signal transduction	GTP binding; GTP-dependent protein binding; Rac GTPase binding	cell cortex; plasma membrane; ruffle
Q6RUV5	Rac protein signal transduction; actin cytoskeleton organization; bone resorption; cell proliferation; cellular component movement; chemotaxis; positive regulation of DNA replication	GTP binding; GTPase activity; protein binding	Golgi membrane; melanosome; plasma membrane
P01887	antigen processing and presentation of …; cellular defense response; immune response; positive regulation of T cell mediated cytotoxicity; response to molecule of bacterial origin	protein binding	external side of plasma membrane; MHC class I protein complex
