# Supplementary material for: Structure Collisions between Interacting Proteins
Source: PLoS One. 2011 Jun 2;6(6):e19581. doi: 10.1371/journal.pone.0019581 (PMC3107212; doi:10.1371/journal.pone.0019581)
Supplement: Table S3 — Protein interactions with single-domain interface. (PDF) [file pone.0019581.s003.pdf]

Table S3. Protein interactions with single-domain interface. 42 interaction pairs are listed whose interface residues fall in a single domain-coding region. The respective Pfam domains are shown in the third and fourth columns. The last columns give the overlap of the secondary proteins according to MSMS and ALPHAVOL and the RMSD of the superimposed primary proteins.

Primary Protein	Protein Name	Domain	Domain Name	Secondary Proteins	Protein Names	Overlap MSMS	Overlap ALPHAVOL	RMSD
P61889	Malate dehydrogenase	PF02866	Ldh_1_C	[P61889, A1AGC9]	[Malate dehydrogenase, Malate dehydrogenase]	8442	16549	0.43
P61889	Malate dehydrogenase	PF02866	Ldh_1_C	[P61889, A1AGC9]	[Malate dehydrogenase, Malate dehydrogenase]	8295	16189	1.56
P02787	Serotransferrin	PF00405	Transferrin	[P02786, P02787]	[Transferrin receptor protein 1, Serotransferrin]	5892	13131	0.60
P02787	Serotransferrin	PF00405	Transferrin	[P02786, P02787]	[Transferrin receptor protein 1, Serotransferrin]	5846	13006	0.60
P62937	Peptidyl-prolyl cis-trans isomerase A	PF00160	Pro_isomerase	[P63098, P62937]	[Calcineurin subunit B type 1, Peptidyl-prolyl cis-trans isomerase A]	5140	10179	0.54
P00459	Nitrogenase iron protein 1	PF00142	Fer4_NifH	[P07328, P07329]	[Nitrogenase molybdenum-iron protein alpha chain, Nitrogenase molybdenum-iron protein beta chain]	5103	13688	1.73
P08160	Early 35 kDa protein	PF02331	P35	[P08160, Q14790]	[Early 35 kDa protein, Caspase-8]	5093	10400	6.89
P00459	Nitrogenase iron protein 1	PF00142	Fer4_NifH	[P07328, P07329]	[Nitrogenase molybdenum-iron protein alpha chain, Nitrogenase molybdenum-iron protein beta chain]	5090	13667	1.73
P00459	Nitrogenase iron protein 1	PF00142	Fer4_NifH	[P07328, P07329]	[Nitrogenase molybdenum-iron protein alpha chain, Nitrogenase molybdenum-iron protein beta chain]	5090	13951	1.74
P00459	Nitrogenase iron protein 1	PF00142	Fer4_NifH	[P07328, P07329]	[Nitrogenase molybdenum-iron protein alpha chain, Nitrogenase molybdenum-iron protein beta chain]	5051	13644	1.74
P62937	Peptidyl-prolyl cis-trans isomerase A	PF00160	Pro_isomerase	[P63098, P62937]	[Calcineurin subunit B type 1, Peptidyl-prolyl cis-trans isomerase A]	4999	9770	0.35
P00459	Nitrogenase iron protein 1	PF00142	Fer4_NifH	[P07328, P07329]	[Nitrogenase molybdenum-iron protein alpha chain, Nitrogenase molybdenum-iron protein beta chain]	4971	13093	1.74
P00459	Nitrogenase iron protein 1	PF00142	Fer4_NifH	[P07328, P07329]	[Nitrogenase molybdenum-iron protein alpha chain, Nitrogenase molybdenum-iron protein beta chain]	4963	13246	1.76
P62937	Peptidyl-prolyl cis-trans isomerase A	PF00160	Pro_isomerase	[P63100, P62937]	[Calcineurin subunit B type 1, Peptidyl-prolyl cis-trans isomerase A]	4938	9847	0.48
P00459	Nitrogenase iron protein 1	PF00142	Fer4_NifH	[P07328, P07329]	[Nitrogenase molybdenum-iron protein alpha chain, Nitrogenase molybdenum-iron protein beta chain]	4933	13580	1.74
P00459	Nitrogenase iron protein 1	PF00142	Fer4_NifH	[P07328, P07329]	[Nitrogenase molybdenum-iron protein alpha chain, Nitrogenase molybdenum-iron protein beta chain]	4927	12821	1.73

P00459	Nitrogenase iron protein 1	PF00142	Fer4_NifH	[P07328, P07329]	[Nitrogenase molybdenum-iron protein alpha chain, Nitrogenase molybdenum-iron protein beta chain]	4921	12797	1.73
P00459	Nitrogenase iron protein 1	PF00142	Fer4_NifH	[P07328, P07329]	[Nitrogenase molybdenum-iron protein alpha chain, Nitrogenase molybdenum-iron protein beta chain]	4847	12725	1.74
P00459	Nitrogenase iron protein 1	PF00142	Fer4_NifH	[P07328, P07329]	[Nitrogenase molybdenum-iron protein alpha chain, Nitrogenase molybdenum-iron protein beta chain]	4816	12833	1.74
P00459	Nitrogenase iron protein 1	PF00142	Fer4_NifH	[P07328, P07329]	[Nitrogenase molybdenum-iron protein alpha chain, Nitrogenase molybdenum-iron protein beta chain]	4772	12541	1.76
P12497	Gag-Pol polyprotein	PF00607	Gag_p24	[Q9JL77, P12497]	[Anti-myosin immunoglobulin heavy chain variable region, Gag-Pol polyprotein]	4411	9049	3.02
P08160	Early 35 kDa protein	PF02331	P35	[P08160, Q14790]	[Early 35 kDa protein, Caspase-8]	4196	9002	6.91
P08160	Early 35 kDa protein	PF02331	P35	[Q14790, P08160]	[Caspase-8, Early 35 kDa protein]	4193	9017	6.88
P00459	Nitrogenase iron protein 1	PF00142	Fer4_NifH	[P07328, P07329]	[Nitrogenase molybdenum-iron protein alpha chain, Nitrogenase molybdenum-iron protein beta chain]	3839	11661	0.74
P01903	HLA class II histocompatibility antigen, DR alpha chain	PF00993	MHC_II_alpha	[P01850, Q48898]	[T-cell receptor beta chain C region, Superantigen]	3002	7813	0.92
P62937	Peptidyl-prolyl cis-trans isomerase A	PF00160	Pro_isomerase	[Q4JL05, P63098]	[Gag polyprotein, Calcineurin subunit B type 1]	2953	6103	0.60
P62937	Peptidyl-prolyl cis-trans isomerase A	PF00160	Pro_isomerase	[Q4JL05, P63098]	[Gag polyprotein, Calcineurin subunit B type 1]	2912	6074	0.60
P62937	Peptidyl-prolyl cis-trans isomerase A	PF00160	Pro_isomerase	[Q4JL05, P63098]	[Gag polyprotein, Calcineurin subunit B type 1]	2872	6027	0.60
P62937	Peptidyl-prolyl cis-trans isomerase A	PF00160	Pro_isomerase	[Q4JL04, P63098]	[Gag polyprotein, Calcineurin subunit B type 1]	2807	5995	0.61
P62937	Peptidyl-prolyl cis-trans isomerase A	PF00160	Pro_isomerase	[Q4JL04, P63098]	[Gag polyprotein, Calcineurin subunit B type 1]	2800	5905	0.61
P62937	Peptidyl-prolyl cis-trans isomerase A	PF00160	Pro_isomerase	[Q4JL04, P63098]	[Gag polyprotein, Calcineurin subunit B type 1]	2760	5962	0.60
P62937	Peptidyl-prolyl cis-trans isomerase A	PF00160	Pro_isomerase	[Q8Q0Z0, P63098]	[Gag polyprotein, Calcineurin subunit B type 1]	2733	5717	0.54
P32851	Syntaxin-1A	PF05739	SNARE	[P32851, Q9GM34]	[Syntaxin-1A, Synaptosomal-associated protein]	2615	7592	1.18
P32851	Syntaxin-1A	PF05739	SNARE	[P32851, P60881]	[Syntaxin-1A, Synaptosomal-associated protein 25]	2567	7373	1.12
P13272	Cytochrome b-c1 complex subunit Rieske, mitochondrial	PF02921	UCR_TM	[P13271, P00157]	[Cytochrome b-c1 complex subunit 8, Cytochrome b]	2434	6434	6.10

P13272	Cytochrome b-c1 complex subunit Rieske, mitochondrial	PF02921	UCR_TM	[P13271, P00157]	[Cytochrome b-c1 complex subunit 8, Cytochrome b]	2425	6440	6.21
P13272	Cytochrome b-c1 complex subunit Rieske, mitochondrial	PF02921	UCR_TM	[P13271, P00157]	[Cytochrome b-c1 complex subunit 8, Cytochrome b]	2399	6203	5.96
P13272	Cytochrome b-c1 complex subunit Rieske, mitochondrial	PF02921	UCR_TM	[P13271, P00157]	[Cytochrome b-c1 complex subunit 8, Cytochrome b]	2371	6469	4.89
P32851	Syntaxin-1A	PF05739	SNARE	[P32851, Q9GM34]	[Syntaxin-1A, Synaptosomal-associated protein]	2359	7016	1.28
P32851	Syntaxin-1A	PF05739	SNARE	[P32851, P60881]	[Syntaxin-1A, Synaptosomal-associated protein 25]	2294	7083	1.94
P32851	Syntaxin-1A	PF05739	SNARE	[P32851, Q9GM34]	[Syntaxin-1A, Synaptosomal-associated protein]	2205	7047	1.65
P32851	Syntaxin-1A	PF05739	SNARE	[P32851, Q9GM34]	[Syntaxin-1A, Synaptosomal-associated protein]	2129	6798	2.11
