# Supplementary material for: Structure Collisions between Interacting Proteins
Source: PLoS One. 2011 Jun 2;6(6):e19581. doi: 10.1371/journal.pone.0019581 (PMC3107212; doi:10.1371/journal.pone.0019581)
Supplement: Table S4 — GO annotations of Pfam domains. (PDF) [file pone.0019581.s004.pdf]

Table S4. GO annotations of Pfam domains. Pfam domains participating in the 42 interaction pairs are listed together with their GO annotations and the number of instances in the 42 single-domain results.

Pfam Domain	Domain Name	GO Annotation	Instances in Single-Domain Results
PF00142	Fer4_NifH	ATP binding; oxidoreductase activity	13
PF00160	Pro_isomerase	peptidyl-prolyl cis-trans isomerase activity; protein folding	10
PF05739	SNARE		6
PF02921	UCR_TM	ubiquinol-cytochrome-c reductase activity	4
PF02331	P35	caspase inhibitor activity; anti-apoptosis	3
PF02866	Ldh_1_C	oxidoreductase activity	2
PF00405	Transferrin	extracellular region; ferric iron binding; cellular iron ion homeostasis; iron ion transport	2
PF00607	Gag_p24	viral reproduction	1
PF00993	MHC_II_alpha	membrane; MHC class II protein complex; antigen processing and presentation; immune response	1
