# Supplementary material for: Structure Collisions between Interacting Proteins
Source: PLoS One. 2011 Jun 2;6(6):e19581. doi: 10.1371/journal.pone.0019581 (PMC3107212; doi:10.1371/journal.pone.0019581)
Supplement: Table S5 — List of colliding protein interaction pairs after filtering with 3D Complex. (PDF) [file pone.0019581.s005.pdf]

Table 5. List of colliding protein interaction pairs after filtering with 3D Complex.

Primary Protein	Protein Name	Secondary Proteins	Protein Names	Instances
P69905	Hemoglobin subunit alpha	[P68871, P69905]	[Hemoglobin subunit beta, Hemoglobin subunit alpha]	150
P69905	Hemoglobin subunit alpha	[P02042, P69905]	[Hemoglobin subunit delta, Hemoglobin subunit alpha]	32
P32851	Syntaxin-1A	[P32851, P60881]	[Syntaxin-1A, Synaptosomal-associated protein 25]	7
P60881	Synaptosomal-associated protein 25	[P32851, P60881]	[Syntaxin-1A, Synaptosomal-associated protein 25]	6
P10912	Growth hormone receptor	[P01241, P10912]	[Somatotropin, Growth hormone receptor]	5
P32851	Syntaxin-1A	[P32851, Q9GM34]	[Syntaxin-1A, Synaptosomal-associated protein]	4
P69905	Hemoglobin subunit alpha	[P02100, P69905]	[Hemoglobin subunit epsilon, Hemoglobin subunit alpha]	2
P68390	Ovomucoid	[P00761, P00766]	[Trypsin, Chymotrypsinogen A]	2
P60881	Synaptosomal-associated protein 25	[P32851, P63027]	[Syntaxin-1A, Vesicle-associated membrane protein 2]	1
P60881	Synaptosomal-associated protein 25	[P60881, Q9N0Y0]	[Synaptosomal-associated protein 25, Vesicle-associated membrane protein 2]	1
P60881	Synaptosomal-associated protein 25	[P32851, Q9N0Y0]	[Syntaxin-1A, Vesicle-associated membrane protein 2]	1
P60881	Synaptosomal-associated protein 25	[P60881, P63045]	[Synaptosomal-associated protein 25, Vesicle-associated membrane protein 2]	1
P60881	Synaptosomal-associated protein 25	[P32851, P63045]	[Syntaxin-1A, Vesicle-associated membrane protein 2]	1
P60881	Synaptosomal-associated protein 25	[P60881, P63027]	[Synaptosomal-associated protein 25, Vesicle-associated membrane protein 2]	1
P62937	Peptidyl-prolyl cis-trans isomerase A	[P63098, Q4JL04]	[Calcineurin subunit B type 1, Gag polyprotein]	1
P62937	Peptidyl-prolyl cis-trans isomerase A	[P63098, Q4JL05]	[Calcineurin subunit B type 1, Gag polyprotein]	1
Q9H1K0	Rabenosyn-5	[P20338, P35285]	[Ras-related protein Rab-4A, Ras-related protein Rab-22A]	1
P63000	Ras-related C3 botulinum toxin substrate 1	[P53365, Q6RUV5]	[Arfaptin-2, Ras-related C3 botulinum toxin substrate 1]	1
P68390	Ovomucoid	[P00761, P08246]	[Trypsin, Leukocyte elastase]	1
